# Taurine protects against As_2_O_3_-induced autophagy in livers of rat offsprings through PPARγ pathway

**DOI:** 10.1038/srep27733

**Published:** 2016-06-13

**Authors:** Jie Bai, Xiaofeng Yao, Liping Jiang, Qiaoting Zhang, Huai Guan, Shuang Liu, Wei Wu, Tianming Qiu, Ni Gao, Lei Yang, Guang Yang, Xiance Sun

**Affiliations:** 1 Department of Occupational and Environmental Health, Dalian Medical University, 9WLvshun South Road, Dalian 116044, PR China; 2 Liaoning Anti-Degenerative Diseases Natural Products Engineering Research Center, Dalian Medical University, 9WLvshun South Road, Dalian 116044, PR China; 3 Department of Obstetrics and Gynecology, NO. 210 Hospital of PLA, Dalian 116021, PR China

## Abstract

Chronic exposures to arsenic had been associated with metabolism diseases. Peroxisome proliferator-activated receptor gamma (PPARγ) was found in the liver, regulated metabolism. Here, we found that the expression of PPARγ was decreased, the generation of reactive oxygen species (ROS) and autophagy were increased after treatment with As_2_O_3_ in offsprings’ livers. Taurine (Tau), a sulfur-containing β–amino acid could reverse As_2_O_3_-inhibited PPARγ. Tau also inhibit the generation of ROS and autophagy. We also found that As_2_O_3_ caused autophagic cell death and ROS accelerated in HepG2 cells. Before incubation with As_2_O_3_, the cells were pretreated with PPARγ activator Rosiglitazone (RGS), we found that autophagy and ROS was inhibited in HepG2 cells, suggesting that inhibition of PPARγ contributed to As_2_O_3_-induced autophagy and the generation of ROS. After pretreatment with Tau, the level of PPARγ was improved and the autophagy and ROS was inhibited in As_2_O_3_-treated cells, suggesting that Tau could protect hepatocytes against As_2_O_3_ through modulating PPARγ pathway.

Arsenic was a ubiquitous naturally occurring metalloid toxicant and carcinogen that found in groundwater, food, dust, and ambient air. It posed health risks to more than 2% of the world population. Chronic arsenic exposure increased the risk of a number of cancers and chronic noncancer diseases, including cardiovascular, pulmonary, and metabolic diseases^1^,^2^. Prenatal arsenic exposure had been associated with altered gene expression in human cord blood leukocytes and various target tissues in rodents^3^. Transplacental studies in mice showed that the offspring of dams who were given, 42.5 ppm and 85 ppm arsenic via drinking water from gestational day 8 to 18 (last two-thirds of pregnancy) had a dose-dependent increase in liver, lung, ovary, and adrenal tumors when they reached adulthood. Furthermore, mice that received arsenic exposure *in utero* and throughout their life course developed more frequent and aggressive tumors at much lower doses compared with mice that only received arsenic exposure during the gestational period^4^. Inorganic arsenicals were converted to monomethylarsenic acid (MMA) and dimethylarsenicacid (DMA) in the body, predominantly in the liver^5^. Therefore, exposure to arsenic might induce hepatotoxicity.

Autophagy occurred in all nucleated type cells and the process of autophagic flux was essential in animal, plant, and yeast cells^6^,^7^. It was a highly regulated lysosomal pathway involved in the recycling of cytosol and the removal of superfluous or damaged organelles. Autophagy was essential for the survival, differentiation, development and homeostasis of cells. Dysregulated autophagy had been suggested to play pathogenic roles in a variety of disease processes including cancer, neurodegeneration, diabetes, aging and heart disease^8^,^9^. In the process of autophagy, some autophagy-related (ATG) family members were transcriptionally induced and the conversion of LC3, a protein involved in autophagosome formation, from LC3-I to LC3-II can be facilitated^10^,^11^. LC3 was an autophagy biomarker that was lapidated during induction of autophagic flux and was required for autophagosome formation. Another autophagy marker was p62/SQSTM1. P62 played an important role in the degradation of polyubiquitinated substrates by autophagy, thus causing its own degradation^12^. Our previous studies had indicated that arsenic increased autophagosome formation and caused autophagic cell death in INS-1 cells^13^. Whether arsenic exerted hepatotoxicity and the precise molecular mechanisms of arsenic hepatotoxicity were not completely elucidated.

The peroxisome proliferator-activated receptors (PPARs) were a family of nuclear fatty acid receptors that regulated tissue specific cellular metabolism and differentiation^14^. In humans, there were three PPAR isoforms, α, β and γ, which respond to a discrete set of ligands^15^. Peroxisome proliferator-activated receptor-γ (PPARγ) was a ligand-activated transcription factor of the nuclear hormone receptor super family. PPARγ was highly expressed in adipocytes and the liver^10^,^14^,^16^. These nuclear receptors when activated directly bind to DNA and regulated gene expression through transcriptional activation (also called master regulators of transcriptional cascades). PPARγ was involved in a variety of physiological processes, including the regulation of the metabolism, inflammation, cellular growth and differentiation^17^,^18^. Previous study had found that the disruption of PPARγ might result the activation of autophagy^19^. In our study, we explored the relationship between PPARγ and the autophagy induced by arsenic in offsprings’ livers.

PPARγ was activated by the thiazolidinediones (TZDs), a group of drugs widely used in patients in the management of type 2 diabetes (T2D), as they regulate glucose metabolism, adiposeness, differentiation, and the expression of several genes including antioxidant defenses^14^,^20^. However, TZDs were once withdrawn from the market or had restricted prescription because they provoked adverse effects such as weight gain, edema, liver injury, cancer, and heart failure^21^. Taurine (Tau), a sulfur-containing β–amino acid, was a major free intracellular amino acid present in many tissues of human and animals^22^. It was mainly distributed in the brain, heart, kidney, and reproductive organs, with many physiological activities^23^. Biosynthesis and dietary intake were the only sources of Tau in our bodies. Tau was synthesized from methionine and cysteine mainly in the liver and the biosynthetic capacity of Tau was very low in human^24^,^25^. Its recognized metabolic function in liver was conjugation with bile acids, which was important for bile secretion and lipid digestion^24^,^26^. Recent studies reported that Tau supplementation could prevent diabetes mellitus, insulin resistance and its complications^9^,^24^. Tau was a key determinant of oxidative phosphorylation and it played an essential role in mitochondria. It was reported that mitochondrial ROS generation was enhanced in taurine-deficient heart^27^,^28^. Nrf2 played a critical role in defending against oxidative stress and inflammation^29^. When oxidative stress was stimulated, Nrf2 would bind to antioxidant response elements (ARE)^30^. Eventually, it led to the restoration of cellular redox homeostasis^31^. The thioredoxin (Trx) system also played the antioxidant role. The cysteine disulfide bridges in oxidized proteins were reduced by Trx system^32^. It was found that PPARγ could up-regulate Nrf2^33^,^34^. Our previous study had found that Tau protected against As_2_O_3_-induced autophagy in pancreas of rat offsprings through Nrf2/Trx pathway^35^. Therefore, we investigated whether Tau executed the action of protecting hepatocytes against As_2_O_3_ through modulating PPARγ/Nrf2 pathway in this study.

## Results

### As_2_O_3_ changed offsprings’ livers morphology

**Figure 1:**
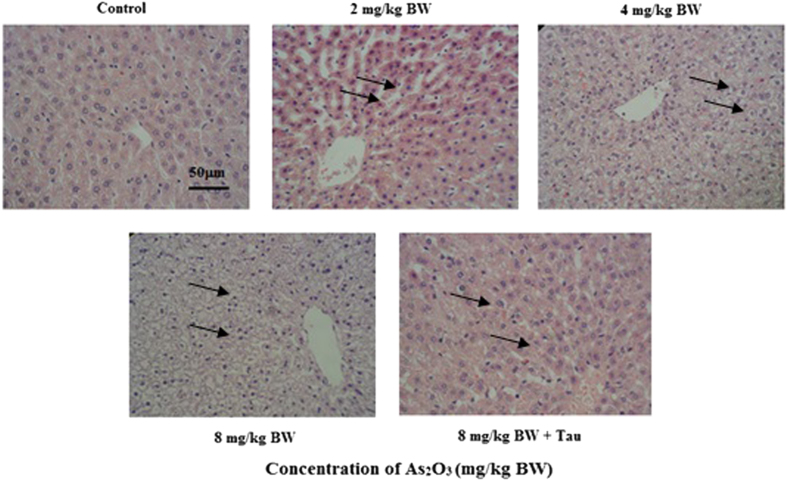
The morphology of As_2_O_3_-treated offsprings’ livers by hematein eosin (scale bar = 50 μM). Arrows indicate hepatic cells.

Pathological analysis showed that the size of hepatocyte was decreased after treatment with As_2_O_3_ (Fig. 1). We found that hepacyte edema occurred in As_2_O_3_-treated offsprings’ livers. The size of As_2_O_3_-treatedhepatocyte was increased and the cellular edema was disappeared after pretreatment with Tau.

### The effects of As_2_O_3_ on offsprings’ livers weight and body weight

The offsprings’ liver weight gains were significantly increased after treatment with As_2_O_3_, but As_2_O_3_ did not affect the body weights (Table 1). After pretreatment with Tau, the liver weights were significantly decreased (Table 1). These changes might be related with the cellular edema in offsprings’ livers.

### As_2_O_3_ caused autophagosome accumulated in livers

**Figure 2:**
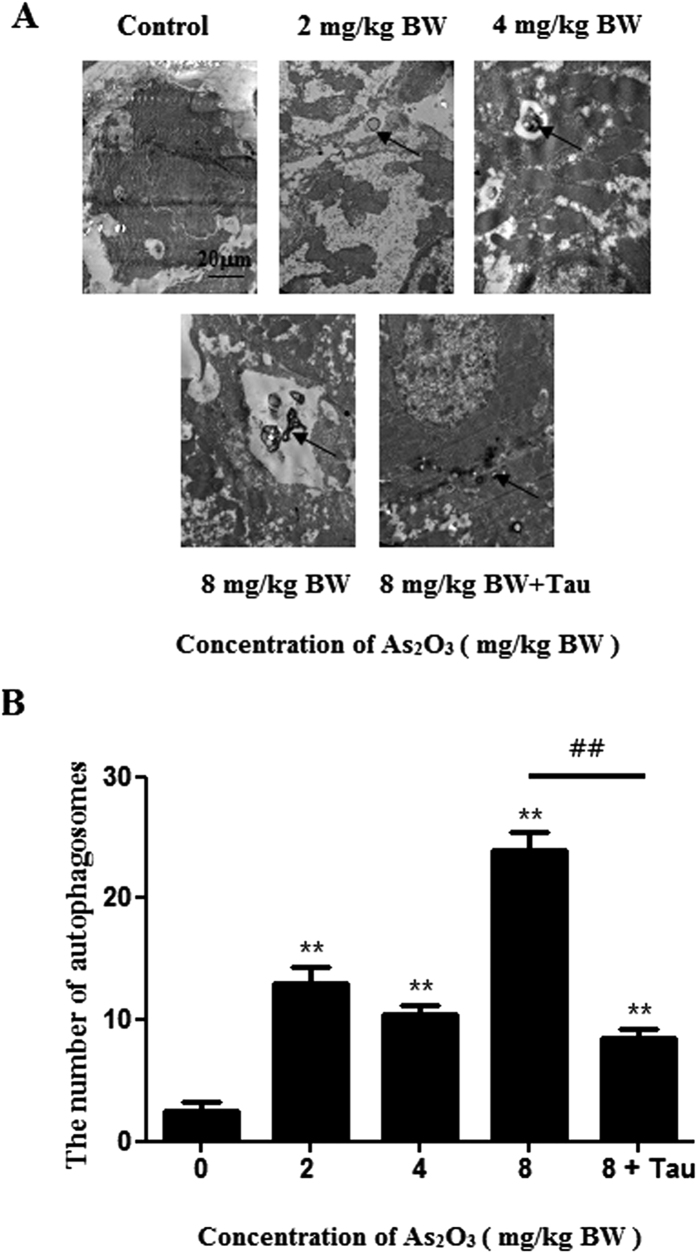
Ultrastructural features of As_2_O_3_-treated offsprings’ livers by transmission electron microscope. (**A**) The rats were treated with 2 mg/kg BW to 8 mg/kg BW As_2_O_3_. The rats of group 5 were pretreated with Tau before treatment with 8 mg/kg BW As_2_O_3._ Arrows indicate autophagosomes (scale bar = 20 μM). (**B**) Comparison of the numbers of autophagosomes per viable cell (n = 15). Bar represents mean ± SD. (**P < 0.01 vs. control; ^##^*P* < 0.01).

To investigate whether autophagy was involved in As_2_O_3_-induced toxicity, we utilized transmission electron microscopy to observe the ultrastructure in As_2_O_3_-treatedlivers. Morphological hallmark of autophagy was the presence of autophagosomes (Fig. 2A). Quantification of the autophagosomes numbers per cell demonstrated that As_2_O_3_ increased autophagosomes number significantly and in a dose-dependent manner. The number of autophagosomes in As_2_O_3_-treated rats was obviously decreased by pretreatment with Tau (Fig. 2B).

### As_2_O_3_ activated autophagy in offsprings’ livers

**Figure 3:**
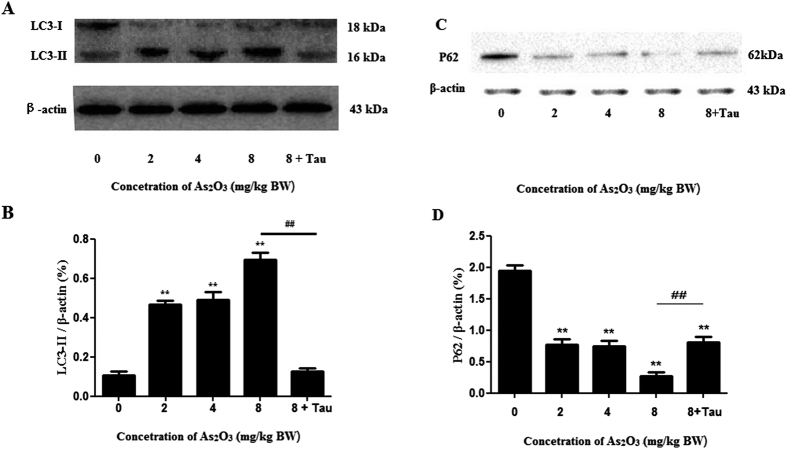
Expression of LC3-II and P62 in As_2_O_3_-treated offsprings’ livers. (**A**) The rats were treated with 2 mg/kg BW to 8 mg/kg BW As_2_O_3_. The rats of group 5 were pretreated with Tau before treatment with 8 mg/kg BW As_2_O_3_. The protein fraction was analyzed by Western blot. β-actin was taken as internal control. (**B**) Densitometric analyses of LC3-II expressed in livers (**P < 0.01 vs. control; ^##^*P* < 0.01). (**C**) Expression of P62 in As_2_O_3_-treated offsprings’ livers. (**D**) Densitometric analyses of P62 expressed in livers (**P < 0.01 vs. control; ^##^*P* < 0.01).

In this study, we evaluated the expression of autophagy biomarkers, LC3-II and P62, by Western blot analysis (Fig. 3A,C). The level of LC3-II was increased dramatically and the level of P62 was decreased in As_2_O_3_-treated livers as shown in Western blot assay. After pretreatment with Tau, the expression of LC3-II was decreased and the expression of P62 was increased dramatically in As_2_O_3_-treated cells (Fig. 3B,D), this gave us a clue to investigate the correspondence of autophagy and cytotoxicity at different As_2_O_3_ concentration and whether Tau could protect against the autophagy induced by As_2_O_3._

### As_2_O_3_ reduced the expression of PPARγ in offsprings’ livers

**Figure 4:**
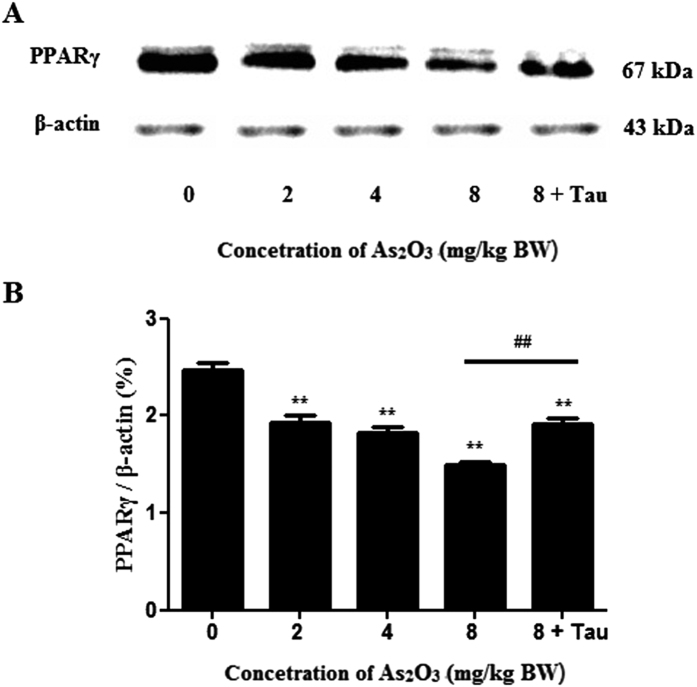
PPARγ protein levels in As_2_O_3_-treated livers. (**A**) The rats were treated with 2 mg/kg BW to 8 mg/kg BW As_2_O_3_. The rats of group 5 were pretreated with Tau before treatment with 8 mg/kg BW As_2_O_3_. The protein fraction was analyzed by Western blot. β-actin was taken as internal control. (**B**) Densitometric analyses of PPARγ levels in offsprings’ livers (**P < 0.01 vs. control; ^##^*P* < 0.01).

PPARγ was a metabolism related protein in livers. The expression of PPARγ was decreased in As_2_O_3_-treated offsprings’ livers (Fig. 4A). Tau could protect against the reduction of PPARγ induced by As_2_O_3_ in offsprings’ livers (Fig. 4B).

### As_2_O_3_ reduced the expression of PPARγ gene in offsprings’ livers

**Figure 5:**
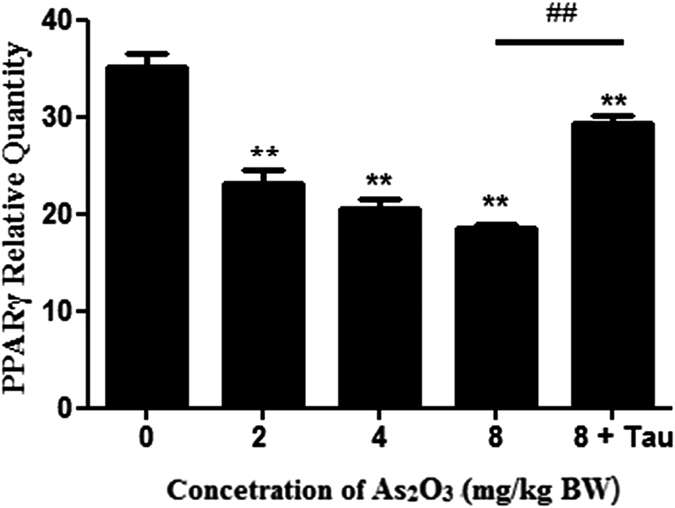
PPARγ gene levels in offsprings’ livers treated with As_2_O_3_. (**A**) The rats were treated with 2 mg/kg BW to 8 mg/kg BW As_2_O_3_. The rats of group 5 were pretreated with Tau before treatment with 8 mg/kg BW As_2_O_3_. The gene fraction was analyzed by RT-PCR. GADPH was taken as internal control. (**B**) Densitometric analyses of PPARγ levels in offsprings’ livers. Relative levels of PPARγ was expressed as a percentage of GADPH (**P < 0.01 vs. control; ^##^*P* < 0.01).

Because of the reduction of PPARγ protein, we used RT-PCR to measure the changes of PPARγ gene levels. The data showed that the expression of PPARγ was decreased significantly in a dose-dependent manner in As_2_O_3_-treated livers (Fig. 5A). After pretreatment with Tau, the expression of PPARγ was accelerated significantly in As_2_O_3_-treated offsprings’ livers (Fig. 5B).

### As_2_O_3_ reduced the expression of Nrf2 and Trx proteins in offsprings’ livers

**Figure 6:**
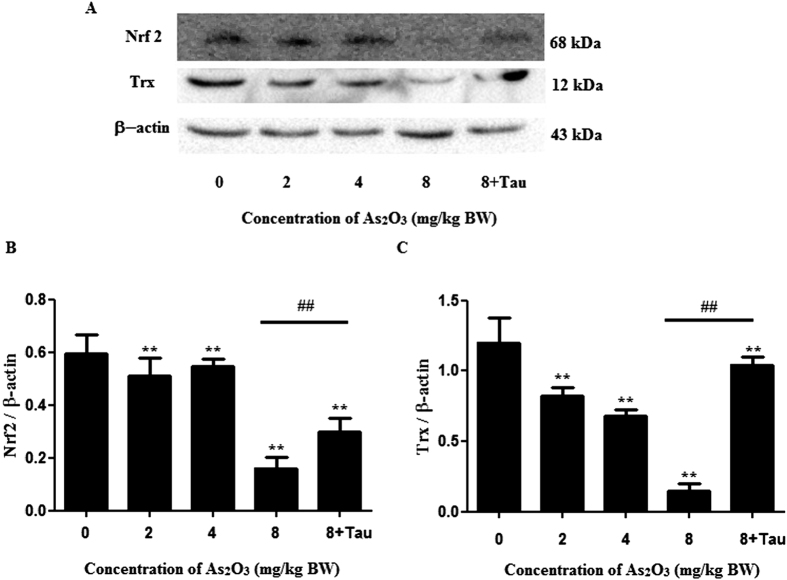
Nrf2 and Trx protein levels in As_2_O_3_-treated livers. (**A**) The rats were treated with 2 mg/kg BW to 8 mg/kg BW As_2_O_3_. The rats of group 5 were pretreated with Tau before treatment with 8 mg/kg BW As_2_O_3_. The protein fraction was analyzed by Western blot. β-actin was taken as internal control. (**B**) Densitometric analyses of Nrf2 levels in offsprings’ livers (**P < 0.01 vs. control; ^##^*P* < 0.01). (**C**) Densitometric analyses of Trx levels in offsprings’ livers (**P < 0.01 vs. control; ^##^*P* < 0.01).

The expression of Nrf2 and Trx proteins were decreased in As_2_O_3_-treated offsprings’ livers (Fig. 6A). Tau could increase the reduction of Nrf2 and Trx induced by As_2_O_3_ in offsprings’ livers (Fig. 6B,C).

### As_2_O_3_ accelerated Malonic Dialdehyde (MDA) level in offsprings’ livers

**Figure 7:**
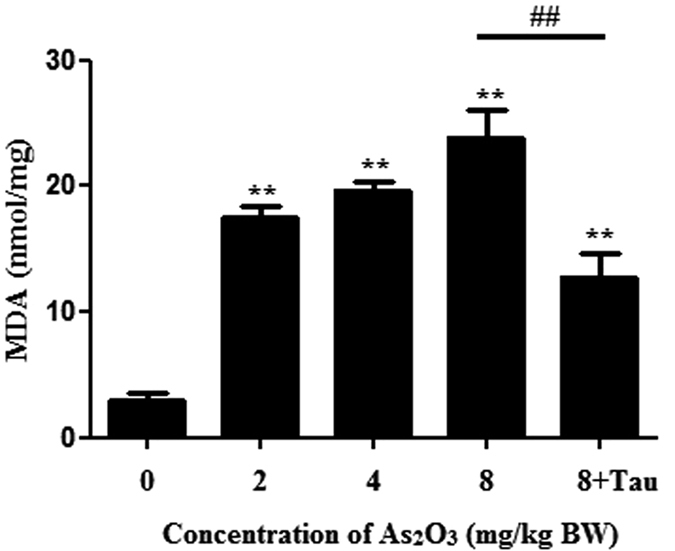
Effects of As_2_O_3_ on MDA level. The rats were treated with 2 mg/kg BW to 8 mg/kg BW As_2_O_3_. The rats of group 5 were pretreated with Tau before treatment with 8 mg/kg BW As_2_O_3_. The level of MDA was measured by MDA test kit (^#^*P* < 0.05; **P < 0.01 vs. control).

As_2_O_3_ could induce autophagy through ROS generation *in vitro*^13^. To investigate the level of oxidative stress, we used MDA test kit to measure the level of MDA. We found that the level of MDA was increased significantly after treatment with As_2_O_3_. After pretreatment with Tau, the level of MDA was decreased in the livers of As_2_O_3_-treated rats (Fig. 7). It suggested that treatment with As_2_O_3_ could accelerate the generation of ROS, and pretreatment with Tau could withstand the oxidative stress induced by As_2_O_3_.

### As_2_O_3_ caused autophagic cell death in HepG2 cells

**Figure 8:**
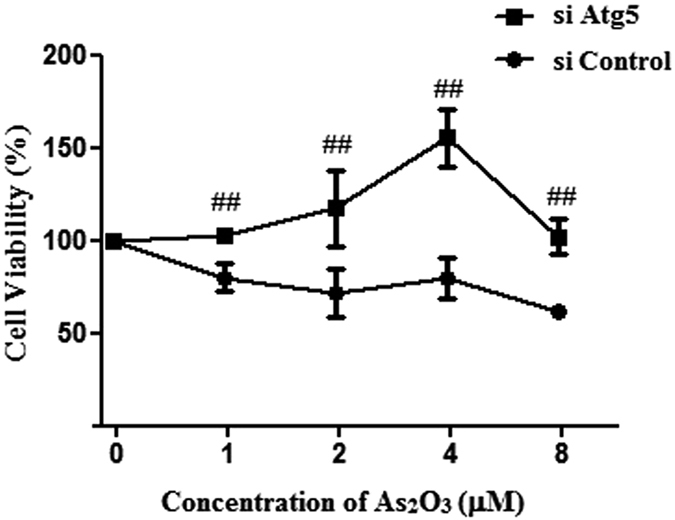
Effect of autophagy on cell viability of As_2_O_3_-treated cells. HepG2 cells were transfected with either 50 nM siRNA against human Atg5 (si Atg5) or scrambled control siRNA (si Control), and then treated with 1 μM–8 μM As_2_O_3_ for 24 h. The cell viability was assessed using the MTT assay. The trace represents the means ± SD. (^##^*P* < 0.01 vs. cells transfected with si Control and treated with the respective As_2_O_3_ concentration, n = 2).

The treatment of HepG2 cells with 1 μM As_2_O_3_ for 24 h resulted in cell death in this study^36^. The cell viability of As_2_O_3_-treated HepG2 cells was increased by knockdown of Atg5 with Atg5 siRNA (Fig. 8). This suggested that autophagy was the major cause of the cell death induced by As_2_O_3_.

### The inhibition of PPARγ contributed to As_2_O_3_-induced cell death

**Figure 9:**
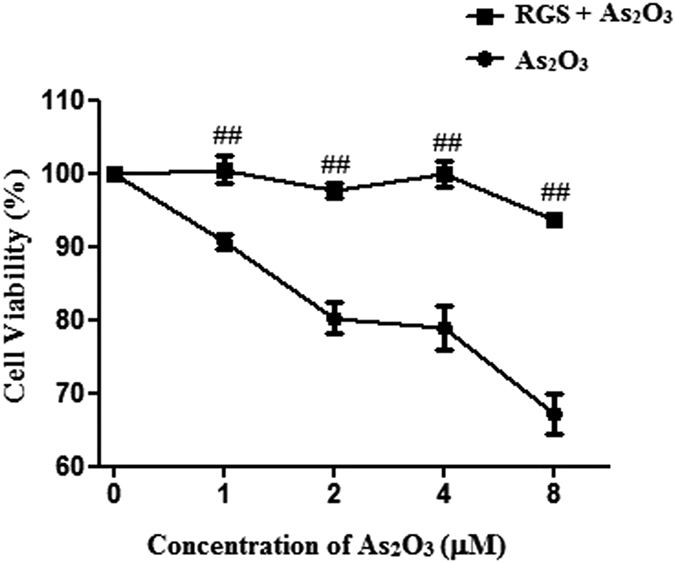
Effect of RGS on the cell viability of As_2_O_3_-treated HepG2 cells. Before incubation with 1 μM As_2_O_3_ for 24 h, the cells were pretreated with 100 μM RGS for 6 h. The cell viability was assessed using the MTT assay. The trace represents the means ± SD. (^##^*P* < 0.01 vs. cells not treatment with RGS and treatment with the respective As_2_O_3_ concentration, n = 2).

The treatment of HepG2 cells with 1 μM As_2_O_3_ for 24 h resulted in cell death in this study. The cell viability of As_2_O_3_-treated HepG2 cells was elevated significantly after pretreatment with RGS, at a concentration of 100 μM (Fig. 9). It suggested that inhibition of PPARγ contributed to As_2_O_3_-induced cell death.

### Tau reduced cell death of As_2_O_3_-treated HepG2 cells

**Figure 10:**
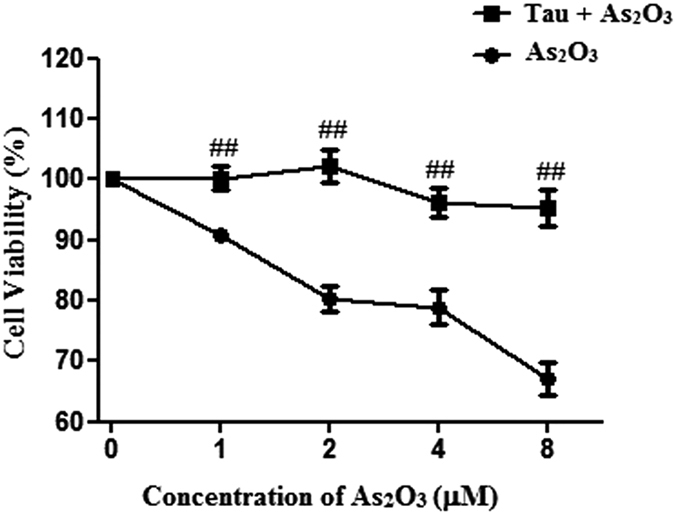
Effect of Tau on the cell viability of As_2_O_3_-treated HepG2 cells. Before incubation with 1 μM As_2_O_3_ for 24 h, the cells were pretreated with 20 μM Tau for 6 h. The cell viability was assessed using the MTT assay. The trace represents the means ± SD. (^##^*P* < 0.01 vs. cells not treated with Tau and treated with the respective As_2_O_3_ concentration, n = 2).

We found that the autophagy in livers was decreased significantly after pretreatment of Tau. To investigate the action of Tau *in vitro*, the As_2_O_3_-treated HepG2 cells were pretreated with Tau at a concentration of 20 μM. The cell viability was elevated significantly (Fig. 10). It suggested that Tau reduced the cell death in As_2_O_3_-treated HepG2 cells.

### RGS and Tau increased the expression of PPARγ in As_2_O_3_–treated HepG2 cells

**Figure 11:**
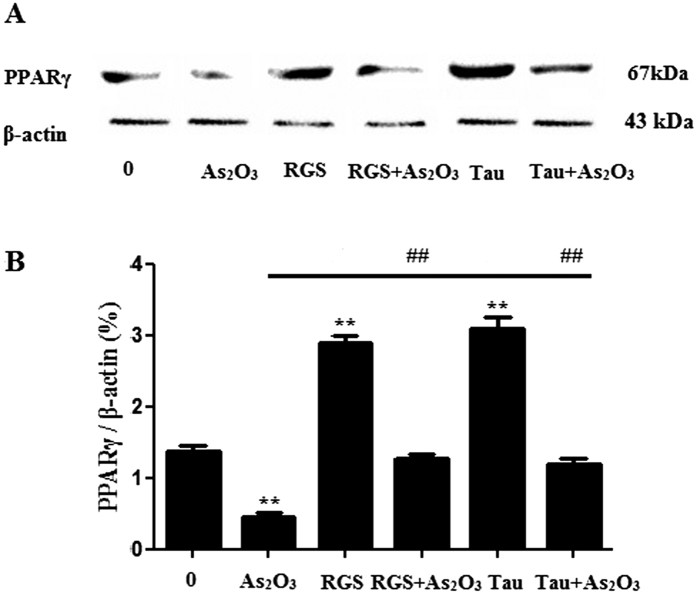
PPARγ protein levels in As_2_O_3_-treated HepG2 cells. (**A**) Before incubation with 1 μM As_2_O_3_ for 24 h, the cells were pretreated with 100 μM RGS and 20 μM Tau respectively for 6 h. The protein fraction was analyzed by Western blot. β-actin was taken as internal control. (**B**) Densitometric analyses of PPARγ levels in HepG2 cells (**P < 0.01 vs. control; ^##^*P* < 0.01).

The expression of PPARγ level was decreased significantly after treatment with 1 μM As_2_O_3_ for 24 h in this study (Fig. 11A). After pretreatment of RGS and Tau respectively, the level of PPARγ was increased significantly in As_2_O_3_-treated HepG2 cells (Fig. 11B).

### The activation of PPARγ and Tau reduced the generation of ROS in As_2_O_3_–treated HepG2 cells

**Figure 12:**
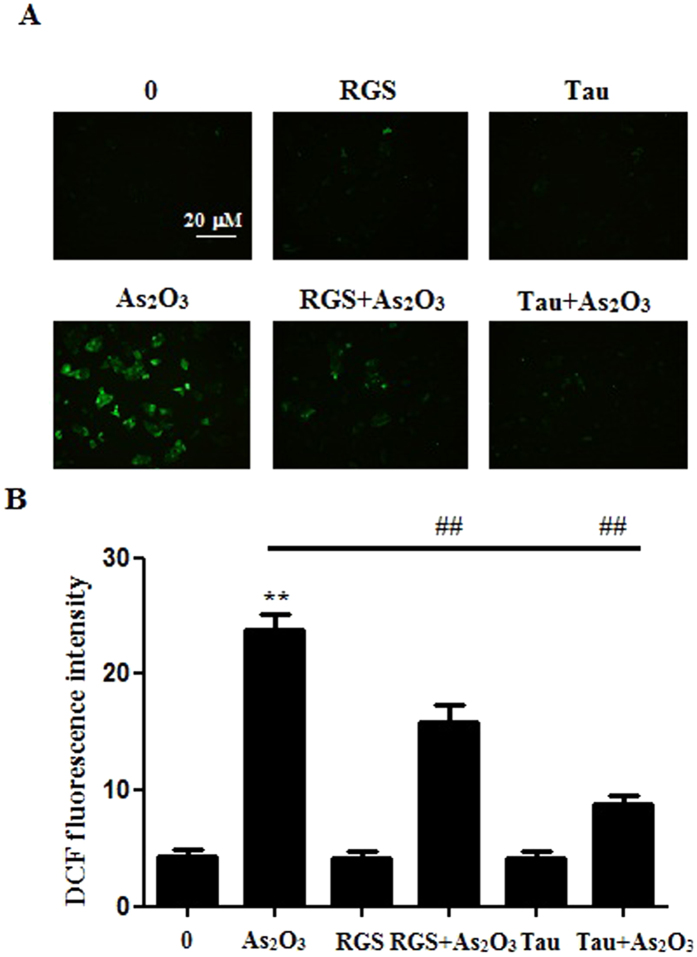
The generation of ROS in As_2_O_3_-treated HepG2 cells. (**A**) Before incubation with 1 μM As_2_O_3_ for 24 h, the cells were pretreated with 100 μM RGS and 20 μM Tau respectively for 6 h. Representative fluorescence microscopic images showing the fluorescence of DCF oxidized by intracellular ROS in HepG2 cells. After treatment, the cells were incubated with 5 μM DCFH-DA for 15 min in the dark and assessed by fluorescence microscope (scale bar = 20 μM). (**B**) The fluorescence intensity of the generation of ROS in As_2_O_3_-treated HepG2 cells (n = 6, **P < 0.01 vs. control; ^##^*P* < 0.01).

The generation of ROS was increased significantly after treatment with 1 μM As_2_O_3_ for 24 h in this study (Fig. 12A). After pretreatment with RGS, the generation of ROS was decreased significantly in As_2_O_3_-treated HepG2 cells (Fig. 12A). It suggested that the activation of PPARγ could reduce the generation of ROS induced by As_2_O_3_. After pretreatment with Tau, the generation of ROS was also decreased significantly in As_2_O_3_-treated HepG2 cells (Fig. 12B). It suggested that Tau had the same effect with RGS. The up-regulation of PPARγ could withstand the generation of ROS induced by As_2_O_3_.

### The activation of PPARγ and Tau accelerated the expression of Nrf2 and Trx proteins in As_2_O_3_-treated HepG2 cells

**Figure 13:**
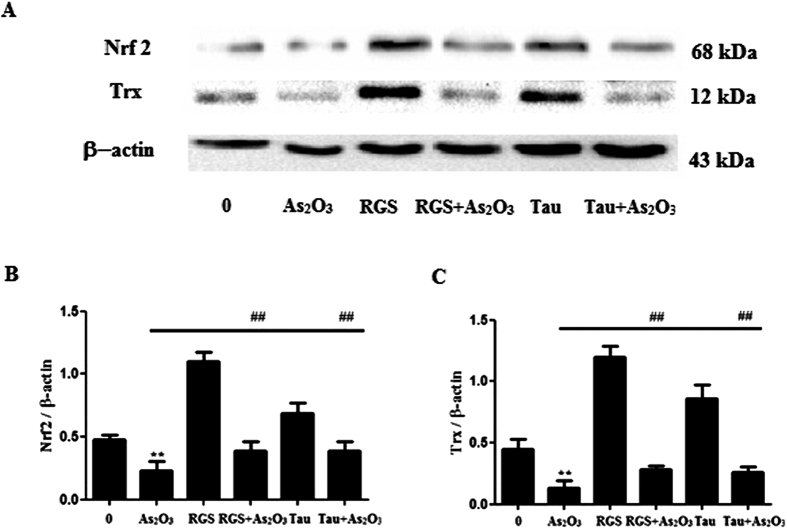
Nrf2 and Trx protein levels in As_2_O_3_-treated HepG2 cells. (**A**) Before incubation with 1 μM As_2_O_3_ for 24 h, the cells were pretreated with 100 μM RGS and 20 μM Tau respectively for 6 h. The protein fraction was analyzed by Western blot. β-actin was taken as internal control. (**B**) Densitometric analyses of Nrf2 protein levels in HepG2 cells (**P < 0.01 vs. control; ^##^*P* < 0.01). (**C**) Densitometric analyses of Trx protein levels in HepG2 cells (**P < 0.01 vs. control; ^##^*P* < 0.01)”.

The expression of Nrf2 and Trx proteins were decreased significantly after treatment with 1 μM As_2_O_3_ for 24 h in this study (Fig. 13A). After pretreatment of RGS, the expression of Nrf2 and Trx were increased significantly in As_2_O_3_-treated HepG2 cells (Fig. 13B,C). It suggested that the activation of PPARγ could accelerate the expression of Nrf2 and Trx. After pretreatment with Tau respectively, the expression of Nrf2 and Trx were also increased significantly (Fig. 13B,C). It suggested that Tau had the effect of up-regulating PPARγ and Tau could withstand the reduction of Nrf2 and Trx induced by As_2_O_3_.

### RGS and Tau reduced autophagy in As_2_O_3_-treated HepG2 cells

**Figure 14:**
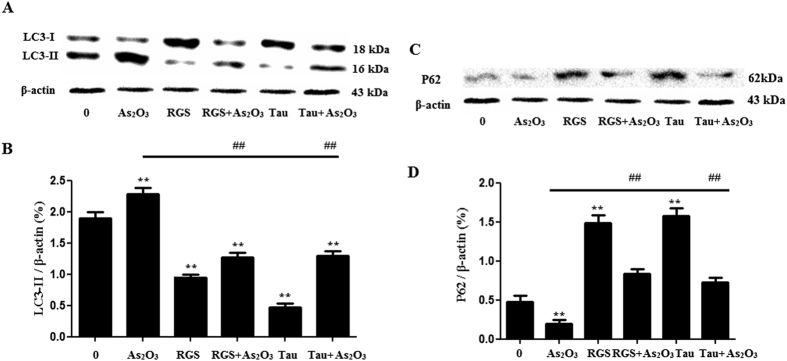
Expression of LC3-II and P62 in HepG2 cells with As_2_O_3_. (**A**) Before incubation with 1 μM As_2_O_3_ for 24 h, the cells were pretreated with 100 μM RGS and 20 μM Tau respectively for 6 h. The protein fraction was analyzed by Western blot. β-actin was taken as internal control. (**B**) Densitometry analyses of LC3-II expressed in As_2_O_3_-treated HepG2 cells (**P < 0.01 vs. control; ^##^*P* < 0.01). (**C**) The expression of P62 in HepG2 cells with As_2_O_3_. Before incubation with 1 μM As_2_O_3_ for 24 h, the cells were pretreated with 100 μM RGS and 20 μM Tau respectively for 6 h. The P62 protein fraction was analyzed by Western blot. β-actin was taken as internal control. (**D**) Densitometry analyses of P62 expressed in As_2_O_3_-treated HepG2 cells (*P < 0.05, **P < 0.01 vs. control; ^##^*P* < 0.01).

In the study, the level of LC3-II was increased significantly and the expression of P62 was decreased in As_2_O_3_-treated HepG2 cells (Fig. 14A,C). After pretreatment with RGS, we found that autophagy was inhibited in As_2_O_3_-treated cells (Fig. 14C,D), suggesting that inhibition of PPARγ contributed to As_2_O_3_-induced autophagy. The level of autophagy was decreased after pretreatment with Tau in As_2_O_3_-treated cells (Fig. 14C,D), suggesting that Tau could protect hepatocytes against As_2_O_3_ through modulating PPARγ–autophagy pathway.

## Discussion

Drinking water contaminated with inorganic arsenic was a major threat to human health with more than 100 million people worldwide exposed to levels that exceed the World Health Organization’s (WHO) recommended limit of 10 μg As/L^37^. Arsenic affected a multitude of biological systems, however, the mechanism by which arsenic elicits its toxic effects remains largely unknown. Numerous studies had been conducted to elucidate the molecular events associated with arsenic exposure, and resulting data suggested multiple mechanisms. Previous studies showed that mice might be less susceptible than human to arsenic toxicity, partly due to a faster metabolism and clearance of arsenic^38^. Therefore, it was necessary to use higher exposure concentration of arsenic than the environmentally relevant concentrations in mouse experiment^39^. The As_2_O_3_ concentration used in this study ranged from 2 mg/kg BW to 8 mg/kg BW.

Autophagy was a gatekeeping mechanism for stabilizing cell homeostasis. Studies had shown that autophagy played important roles in physiology and pathophysiology^40^. Our previous study found that arsenic accelerated autophagosome formation and caused autophagic cell death in INS-1 cells^13^. Autophagy had recently been shown to be involved in metabolism, triggering great interests in elucidating the underlying mechanism and testing the feasibility of targeting autophagy for the prevention and treatment of obesity and related metabolic disorders^41^. In addition to LC3-II, P62 was another autophagy specific substrate. P62 was degraded in autolysosomes^42^. In this study, Western blot analysis revealed that As_2_O_3_ accelerated the expression of LC3-II in offsprings’ livers and the level of P62 was decreased significantly in HepG2 cells after treatment with As_2_O_3_. It was a reliable indicator for the activation of autophagy in As_2_O_3_-treated offsprings’ livers. As_2_O_3_-induced autophagy was prevented by Tau, suggesting that As_2_O_3_-induced injury of livers might have the connection with autophagy and Tau might prevent livers from As_2_O_3_-induced autophagy.

PPARγ, which belonged to a family of nuclear hormone receptors that regulated the function and expression of complex gene networks, especially involved in cell proliferation and differentiation, glucose metabolism and homeostasis, insulin sensitivity and lipid metabolism^43^. It was reported that after treatment with RSG observably attenuated GCI-induced elevation of the LC3-II and Beclin-1 hippocampus of brain, these observations suggested that RSG might exert its inhibitory effect by inactivating neuronal autophagy through decreasing Beclin-1 and LC3-II, and thus achieve a cerebral protective effect^44^. Prenatal arsenic exposure had been associated with altered gene expression in human cord blood leukocytes and various target tissues in rodents^3^. We found that As_2_O_3_ reduced the expression of PPARγ protein and inhibited the expression of PPARγ gene in offsprings’ livers. It suggested that the reduction of PPARγ expression was involved in As_2_O_3_-induced autophagy in offsprings’ livers. After pretreatment with RGS, the expression of PPARγ was increased dramatically and the level of autophagy was decreased in HepG2 cells. It suggested that the inhibition of PPARγ contributed to As_2_O_3_-induced autophagy.

Arsenic was able to induce the generation of ROS^12^. In our previous studies, we found that arsenic could induce autophagic cell death through ROS pathway. Nrf2 was the key factor of the oxidative stress reaction. The Trx system was composed of NADPH, TrxR and Trx. It was also a crucial line of defense against ROS through its activity of disulfide reductase^45^. In this study, we found that after treatment with As_2_O_3_, the expression of Nrf2 and Trx were decreased obviously. The level of MDA was increased significantly in As_2_O_3_-treated livers. We used the activator of PPARγ to investigate whether the up-regulation of PPARγ could accelerate the expression of Nrf2 and Trx. We found that after pretreatment with RGS, the levels of Nrf2 and Trx were both increased obviously. It suggested that the inhibitor of PPARγ contributed to ROS-dependent autophagic injury in As_2_O_3_-treated offsprings’ livers.

It was reported that Tau was a non-essential free amino acid, which was one of the chemical components abundantly present in Lyciumbarbarum and crosses the blood–retinal barrier. A dietary source of Tau was essential for those animals (e.g. cat and humans), which cannot synthesize sufficient Tau and where greater consumption of Tau was required, such as in diabetes. Furthermore, several studies had reported that Tau potentiates the effect of insulin and possibly affected the insulin receptor. In addition, one study had indicated that high concentrations (20 mM) of Tau are capable of enhancing the phosphatidylinositide 3 (PI3)-kinase/Akt signaling pathway responsible for insulin-mediated stimulation of glucose transporter activity and glucose uptake^9^,^16^,^26^. Tau recognized metabolic function in liver was conjugation with bile acids, which was important for bile secretion and lipid digestion^46^. Tau supplementation might prevent alterations or restore endocrine pancreatic mass in malnutrition, obesity, T1D and T2D^9^. In this study, Tau prevented the stimulation of autophagy and the inhibition of PPARγ in As_2_O_3_-treated offsprings’ livers. Tau also reduced the generation of ROS by accelerating the expression of Nrf2 and Trx. The expression of PPARγ was improved dramatically and the level of autophagy was decreased after pretreatment of Tau in As_2_O_3_-treated HepG2 cells. After pretreatment with Tau, the generation of ROS was decreased obviously. It suggested that Tau could accelerate the expression of PPARγ and withstand the hepatotoxicity induced by As_2_O_3_.

## Conclusion

In this study, we found that arsenic induced autophagy in offsprings’ livers through ROS pathway. The reduction of PPARγ level played a crucial role in this process. Tau could reverse arsenic-inhibited PPARγ. Tau could inhibit the generation of ROS and autophagy in arsenic-treated offsprings’ livers. We found that As_2_O_3_ caused autophagic cell death through ROS pathway in HepG2 cells. The inhibition of PPARγ contributed to As_2_O_3_-induced autophagy and generation of ROS. Tau could protect hepatocytes against As_2_O_3_ hepatotoxicity through modulating PPARγ–ROS-autophagy pathway.

## Methods

### Ethics statement

The Animal Ethics Committee of the Institute of Zoology, Dalian Medical University, approved this study. The institute issued an ID [SCKK (Liao) 2002–002] to this animal study and the ethical committee guided the animal use and conduct. All the experimental methods were carried out in accordance with the approved guidelines.

### Experimental groups

To investigate the effects of As_2_O_3_ on offspring, groups of five healthy adult 90 Wistar rats (230 g–250 g) were purchased from Model Animal Research Center of Dalian Medical University (China). Experimental manipulation were described in our previous study. Briefly, the pregnant rats were randomly divided into five groups, each of 10 animals:

Group 1: Control group. Rats were treated with distilled water. The offsprings were treated as their mothers.

Group 2: Rats were treated with 2 mg/kg BW As_2_O_3_. The offsprings were treated as their mothers.

Group 3: Dams were treated with 4 mg/kg BW As_2_O_3_. The offsprings were treated as their mothers.

Group 4: Dams were treated with 8 mg/kg BW As_2_O_3_. The offsprings were treated as their mothers.

Group 5: Dams were treated with 8 mg/kg BW As_2_O_3_ and 150 mg/kg Tau. The offsprings were treated as their mothers.

The rats were given by gavage once a day from GD 6 until PND 42.

### Cell culture and treatment

Human heptoma cell line HepG2 was purchased from the American Type Culture Collection. HepG2 cells were cultured in MEM/EBSS (Hyclone) medium supplemented with 10% fetal bovine serum (Biological Industries) and antibiotics (100 U/ml penicillin and 100 μg/ml streptomycin, Sigma) under a humidified atmosphere with 5% CO_2_ at 37 °C. As_2_O_3_ was purchased from Sigma Aldrich (CAS#: 1327-53-3), and 3.96 mg of As_2_O_3_ was dissolved in 1 ml phosphate buffer saline to prepare a stock solution of 20 μM. The cells were pretreated with 20 μM Tau and 100 μM RGS for 6 h.

### Pathological analysis

Parts of liver were taken and fixed in 10% formalin solution. After 24 h–28 h, the livers were dehydrated in a grade alcohol series and embedded in paraffin wax. Sections of 4 mm–5 mm thickness were stained with hematoxylin-eosin and taken photos by microscope for pathological analysis.

### Transmission electron microscopy

Parts of liver were fixed with 2% glutaraldehyde for 2 h, and then post fixed in 1% osmium tetroxide for 1 h. Dehydration was done in increasing concentration of ethanol followed by propylene oxide. While incubated in 70% ethanol, the pellet was stained en bloc with 1% uranyl acetate. Finally the pellet was embedded in Epon resin. Ultrathin sections were post stained with uranyl acetate and Reynold’s lead citrate routinely. Electron micrographs were taken with JEM 1400 transmission electron microscope at 80 kV.

### Western blot

At the end of the designated treatments, the tissues were washed twice with ice-cold PBS and completely lysed in lysis buffer of a protein extraction kit (Keygen Biotech). The tissues lysate was centrifuged at 14000 *rpm* for 15 min at 4 °C, and the supernatant containing the total protein was collected. The concentration of total protein was quantified using BCA method. SDS-polyacrylamide gel electrophoresis was performed, and the proteins were then transferred to a nitrocellulose membrane. After blocking with 10% non-fat milk, the blots were incubated with primary antibodies against LC3-II (Sigma), PPARγ (Proteintech), Nrf2 (Proteintech), Trx (Abcam) or internal control β-actin (Santa). Blots were then incubated with horseradish peroxidase (HRP)-conjugated secondary antibodies (Sigma) followed by detection with a SuperSignal West Pico Kit (Thermo Scientific) according to the manufacturer’s instructions. The expected protein bands were detected using Bio-Rad ChemiDoc^TM^MP imaging system. Relative abundance was measured with Gel-Pro Analyzer 4.0 software. The results were representative of three independent experiments.

### RT-PCR

Total RNA was isolated using RNAiso Plus (TaKaRa). RT-PCR was performed using PrimeScript^™^ RT reagent Kit (TaKaRa) and Trans Start Top Green Qpcr Super Mix (TRANSGEN BIOTECH). Relative expression of target gene was calculated with ΔΔCT method.

### MTT assay

The cytotoxicity of As_2_O_3_ was detected by MTT assay. HepG2 cells (1 × 10^5^/ml) were seeded in 96-well plates and treated with 1 μM-8 μM As_2_O_3_ for 24 h. After treatment, 3-(4,5-dimethylthiazol-2-yl)-2,5-diphenyltetrazolium bromide (MTT) was added, and the cells were incubated for 4 h at 37 °C. The supernatant was discarded, and 1% DMSO was added. The plate was gently agitated until the blue formazan crystals were fully dissolved. The absorbance at 570 nm was read using a Bio-Rad Microplate Reader, and the cell viability (%) was calculated using the following equation: (A570 of treated sample/A570 of control) × 100.

### RNA interference

HepG2 cells were transfected with either 50 nM siRNA against Atg5 or scrambled control siRNA (Gene Pharma) using transfection reagent Lipofectamine 2000 (Invitrogen) according to the manufacturer’s instructions.

### MDA assay

Oxidative stress was determined by the measurement of MDA^47^. Parts of livers were homogenized in phosphate buffer. After centrifugation, the supernatants were collected for MDA analysis. The level of MDA was quantified using MDA test kit (Keygen Biotech). The MDA concentration was calculated from the absorption at 532 nm.

### Measurement of intracellular ROS

Intracellular ROS generation was detected by fluorescence microscopy, using DCFH-DA. Before incubation with 1 μM As_2_O_3_ for 24 h and were pretreated with RGS and Tau for 6 h. The cells were incubated with DCFH-DA (Dichlorodihydrofluorescein Diacetate, Sigma) at a final concentration of 5 μM for 15 min in the dark, and then viewed under the fluorescence microscope (Olympus BX63). Inside cells, DCFH-DA was oxidized to fluorescent compound DCF by ROS. The intensity of DCF fluorescence was quantified by Image-Pro Plus 7.0 software.

### Statistical analysis

The data were expressed as the means ± standard deviation (SD) from at least three independent experiments performed in triplicate and analyzed using the SPSS 13.0 statistical software. The comparisons between groups were analyzed using one-way ANOVA and Student–Newman–Keuls (SNK) test, and *P* < 0.05 was considered statistically significant.

## Additional Information

**How to cite this article**: Bai, J. *et al*. Taurine protects against As_2_O_3_-induced autophagy in livers of rat offsprings through PPARγ pathway. *Sci. Rep.* **6**, 27733; doi: 10.1038/srep27733 (2016).

## Figures and Tables

**Table 1:** Effects of As_2_O_3_ (2 mg/kg BW to 8 mg/kg BW) on liver and body weights at postnatal day 42 (PND 42) in offspring rats.

**Treatment**	**Body weight (g)**	**Liver weight (g)**
0	228.37 ± 14.32	6.83 ± 0.55
2	232.56 ± 17.08	7.23 ± 0.65*
4	239.87 ± 16.62	7.95 ± 0.03*
8	241.03 ± 17.12	8.27 ± 0.51*
8+Tau	232.13 ± 8.69	7.35 ± 0.07#

Values are means ± SD (n = 7). *p < 0.05 vs control; ^#^*p* < 0.05 vs 8 mg/kg BW.
